# Development of alternative herbals remedy for gastric cancer based on transcriptomic analysis of immune infiltration and ferroptosis

**DOI:** 10.3389/fgene.2023.1086368

**Published:** 2023-03-03

**Authors:** Mingyue Li, Jie Tao, Rui Qian, Feng Jiang, Yinzhi Song, Zhicong Zeng, Changlong Cai

**Affiliations:** ^1^ Department of Pharmacy, Shenzhen Bao’an Chinese Medicine Hospital, Guangzhou University of Chinese Medicine, Shenzhen, Guangdong, China; ^2^ Department of Cardiology, Shenzhen Bao’an Chinese Medicine Hospital, Guangzhou University of Chinese Medicine, Shenzhen, Guangdong, China; ^3^ Department of Gastroenterology, Shenzhen Bao’an Chinese Medicine Hospital, Guangzhou University of Chinese Medicine, Shenzhen, Guangdong, China; ^4^ Department of Surgery, Shenzhen Bao’an Chinese Medicine Hospital, Guangzhou University of Chinese Medicine, Shenzhen, Guangdong, China

**Keywords:** immune infiltration, ferroptosis, gastric cancer, herbal medicine, herbal ingredient

## Abstract

**Objective:** Screening out potential herbal medicines and herbal ingredients for the treatment of gastric cancer based on transcriptomic analysis of immune infiltration and ferroptosis.

**Methods:** Gene expression profiles of gastric tumour tissues and normal tissue samples were obtained from the GEO database and the samples were analysed for immune cell infiltration condition and differential expressed genes of ferroptosis. Key genes were screened by protein-protein interaction (PPI) and enrichment analysis, and molecular docking was used to predict and preliminary validate potential herbal and traditional Chinese medicine components for gastric cancer based on the key genes. Finally, RT-QPCR was used to validate the prediction results.

**Results:** Immune cell infiltration analysis revealed high levels of infiltration of activated CD4 memory T cells, monocytes, M0 macrophages in gastric tumor tissues, while plasma cells and resting mast cells had higher levels of infiltration in the paraneoplastic tissues. Differential gene expression analysis identified 1,012 upregulated genes and 880 downregulated genes, of which 84 immune related differentially expressed genes such as CTSB, PGF and PLAU and 10 ferroptosis-related differentially expressed genes such as HSF1, NOX4 and NF2 were highly expressed in gastric cancer tissues. The results of enrichment analysis showed that they mainly involve 343 biological processes such as extracellular matrix organization and extracellular structural organization; 37 cellular components such as complexes of collagen trimer and basement membrane; 35 molecular functions such as signal receptor activator activity and receptor ligand activity; 19 regulatory pathways such as cytokine-cytokine receptor interactions and retinol metabolism. Finally, two key genes, TLR4 and KRAS, were selected and 12 herbal medicines such as *Radix Salviae liguliobae, Rhizoma Coptidis, Rhizoma Polygoni cuspidati* and 27 herbal ingredients such as resveratrol, salvianolic acid b were predicted on the basis of key genes. Molecular docking results showed that KRAS binds tightly to coumarin and magnolol, while TLR4 can bind tightly to resveratrol, curcumin, salvianolic acid b, shikonin. Subsequently, the effect of resveratrol and magnolol was experimentally verified.

**Conclusion:** Herbal medicines such as *S. liguliobae, Rhizoma Coptidis, Rhizoma P. cuspidati* and herbal ingredients such as resveratrol, curcumin, salvianolic acid b may provide research directions and alternative therapeutic approaches for immunomodulation of TME and ferroptosis of tumour cells in gastric cancer.

## 1 Introduction

Gastric cancer is the fifth most common cancer and the third most common cause of cancer death worldwide (Thrift and El-Serag, 2020), is a global health problem with more than one million new diagnoses each year (Smyth et al., 2020). Although the incidence and mortality rate of gastric cancer has declined over the past half century, it remains the third leading cause of cancer death worldwide (Sexton et al., 2020). Although the 5-year survival rate for patients who undergo surgery at an early stage can be as high as 95%, the recurrence rate after surgery is high (Song et al., 2017). Clinical problems are prominent due to the low rate of early diagnosis of gastric cancer, the large number of patients with advanced stages and the fact that survival time for patients with advanced metastases is usually less than 1 year (Johnston and Beckman, 2019; Tan, 2019; Patel and Cecchini, 2020). A significantly higher proportion of microsatellite instability (MSI) features are present in gastrointestinal adenocarcinomas compared to all other forms of cancer (Liu et al., 2018). According to the Cancer Genome Atlas Research Network, gastric adenocarcinomas can be divided into four distinct subtypes based on the molecular markers that distinguish them: Epstein–Barr virus (EBV) positive tumors, chromosomal instability (CIN), genomically stability, and MSI-H (Liu et al., 2018; Pietrantonio et al., 2019). MSI-H tumors constitute approximately 23 percent of all GC.

Systemic immunotherapy, surgery, targeted therapy, radiotherapy and chemotherapy have all proven effective in the treatment of gastric adenocarcinoma. However, their clinical efficacy has been disappointing, making the pursuit of better treatment strategies a natural choice. Recently, immunotherapy has emerged as a promising approach for the treatment of gastric cancers (GC) (Coutzac et al., 2019). Inhibition of the programmed death-1 (PD-1)/programmed death ligand 1 (PD-L1) axis with immune checkpoint inhibitors (ICIs) such as nivolumab and pembrolizumab has emerged as a novel therapeutic strategy for advanced GC (Abozeid et al., 2017; Bizzaro et al., 2018; Kono et al., 2020). One of the main causes of uncontrolled proliferation of cancer cells is the disturbance of the tumour microenvironment (TME), which contains a large number of negative immune cells that can directly or indirectly create a survival environment favorable to tumor cell proliferation by modulating the immune system (Oya et al., 2020). Cumulative alterations in genes leading to the transformation of normal cells into cancer cells are reflected in next-generation sequencing technologies. Accurate and timely DNA repair is essential to maintain gene stability. Among the many DNA repair pathways, the mismatch repair (MMR) pathway is critical. Lack of MMR contributes to the molecular signature of MSIs, which in turn increases the risk of cancer. Biomarkers, in particular MSI, PD-L1, tumor mutation burden, Epstein-Barr virus, and human epidermal growth factor receptor 2 (HER2) are becoming an increasingly important factors in systemic therapeutic methods and in determining which patient groups are most likely to benefit from immunotherapy and targeted therapies (Joshi and Badgwell, 2021). Ferroptosis is a form of controlled cell death that is dependent on the presence of iron. It is caused by the accumulation of harmful lipid peroxides on cellular membranes (Dixon et al., 2012). This unique form of cell death that does not dependent on programmed regulation and poses a great threat to tumour cells. In recent years, significant progress has been made in understanding the role of ferroptosis in tumour biology and cancer therapy (Hassannia et al., 2019; Mou et al., 2019; Su et al., 2020). As ferroptosis has been increasingly studied, a growing number of findings suggest that ferroptosis frequently interferes with the immune response, leading to inflammation-associated immunosuppression and thus promoting tumour cell growth (Friedmann Angeli et al., 2019; Chen et al., 2021a).

Research models for predicting alternative candidates of Chinese medicine or herbs for the treatment of myocardial infarction (Jiang et al., 2022a), atrial fibrillation (Jiang et al., 2022b) and sports injury (Jiang et al., 2022c) with herbs or Chinese herbal medicines have been developed by us. This research aims to provide a new research mode to predict alternative candidates for the treatment of gastric cancer on the basis of immune infiltration and ferroptosis so that developing new alternative treatment strategies.

## 2 Materials and methods

### 2.1 Gene expression profiles of gastric cancer and normal tissue

In order to obtain gene expression profiles of gastric cancer and normal tissue, the dataset (GSE13911) was searched in GEO database (Table 1), and gene IDs were converted into gene symbols.

**TABLE 1:** All relevant software and websites used in this study.

Name	Entrance
GEO database	https://www.ncbi.nlm.nih.gov/geo/
R soft and main plug-in package	Version: R 4.1.1; Package: limma, clusterprofiler
ImmPort database	https://www.immport.org/home
String databse	https://cn.string-db.org/
Cytoscape	Version: Cytoscape_v3.9.0; Plug-in: Degree
HERB database	http://herb.ac.cn/
PubChem database	https://pubchem.ncbi.nlm.nih.gov/
ChemOffice	Chem3D 19.0
Uniprot database	https://www.uniprot.org/
PDB database	https://www.rcsb.org/
Autodock vina	Autodock vina 1.1.2

### 2.2 Analysis of immune cell infiltration and differentially expressed genes

An analysis of immune cell infiltration was performed using the CIBERSORT deconvolution method (perm = 1,000). Based on the cut-off criteria |logFC| ≥ 1 and adjP ≤ 0.05, gene expression profiles were filtrated for differentially expressed genes (DEGs).

### 2.3 Differential expression of immune-and ferroptosis-related genes

Additionally, we investigated the differential expression of immune- and ferroptosis-related genes among gastric cancer tissue. From the ImmPort and FerrDb databases (Table 1), we identified genes associated with immunity and ferroptosis. Then, by intersecting them with DEGs, we were able to identify Immune-related DEGs (ImmDEGs) as well as ferroptosis-related.

### 2.4 Protein-protein interaction analysis and enrichment analyses

In our study, DEGs were subjected from the STRING database (Table 1). We applied Protein-protein interaction (PPI) analysis to DEGs and screened the top 100 genes using the Cytohubba plugin in Cytoscape. These genes were identified as hub genes. In addition, DEGs were analyzed using the R package clusterprofiler (cutoff: *p* ≤ 0.05 and q ≤ 0.05) for pathway enrichment using Gene Ontology (GO) and Kyoto Encyclopedia of Genes and Genomes (KEGG).

### 2.5 Identification of key genes and prediction of herbal medicine

By intersecting ImmDEG, FerDEG, and hub genes, key genes were identified. A reverse prediction algorithm was used to predict target herbal medicines and ingredients from the HERB database (Table 1) on the basis of key genes.

### 2.6 Molecular docking

We downloaded the protein structure of key genes encoded from PDB database (Table 1), and the structure of predicted herbal ingredients from Pubchem database. Molecular docking of key genes with herbal ingredients method was utilizing Autodock vina tools, which displayed the best bond way for docking the key genes with herbal ingredients with the lowest binding free energy. The lowest bound free energy model was considered to be the best bond path.

### 2.7 Experimental design

The THP-1 cell used to construct the immune infiltration model, The THP-1 cell was purchased from Wuhan (Procell, China), used for *in vitro* research and cultivated in RPMI-1640 medium containing 10% foetal bovine serum (Gibco, MA, United States) and 0.05 mM β- Mercaptoethanol in a 37°C cell incubator with 5% CO_2_. Prior to each experiment, 1.5*10^6^/mL THP-1 cells were inoculated in six-well plates and treated with 100 ng/mL Phorbol-12-myristate-13-acetate (PMA) for 24 h to induce differentiation into macrophages. PMA-induced THP-1 cells were treated as described below. Normal control group (NC): PMA-induced THP-1 cells were cultured in RPMI-1640 medium for 48 h. LPS + INF-Y group: PMA-induced THP-1 cells were incubated with 100 ng/mL lipopolysaccharide (LPS) and 20 ng/mL IFN-γ for 48 h. LPS + INF-Y + Resveratrol: THP-1 cell was first incubated with 2.5 uM Resveratrol for 2 h. LPS + INF-Y+ magnolol: HL-1 was first incubated with 15 uM magnolol for 2 h.

### 2.8 QT-PCR validation

TRIzol reagent was used to extract the total RNA from THP-1 cells. With the use of the EvoM-MLV kits, cDNA was created. On a CFX96 Real-Time PCR Detection System (Bio-Rad Laboratories), quantitative reverse transcription-PCR (RT-qPCR) was carried out using 2X SYBR Green qPCR Master Mix (K1070500, APExBIO, US) as directed by the manufacturer and evaluated using the 2^−ΔΔCT^ method. The ideal temperature conditions were 95°C for 30 s, 95°C for 5 s, and 40 cycles at 60°C for 5 s. The expression of the target genes was examined using the 2^−ΔΔCT^ technique after the amounts of mRNA were adjusted to endogenous GAPDH.

## 3 Results

### 3.1 Gene expression profiles

From the GEO database, the targeted gene expression matrix (GSE13911) attained by our team. In the data matrix, gene expression levels are compared between gastric tumor tissue and normal tissue. The gastric cancer group included 38 samples, and the normal tissue group included 31 samples.

### 3.2 Analysis of immune cell infiltration and DEGs

**FIGURE 1:**
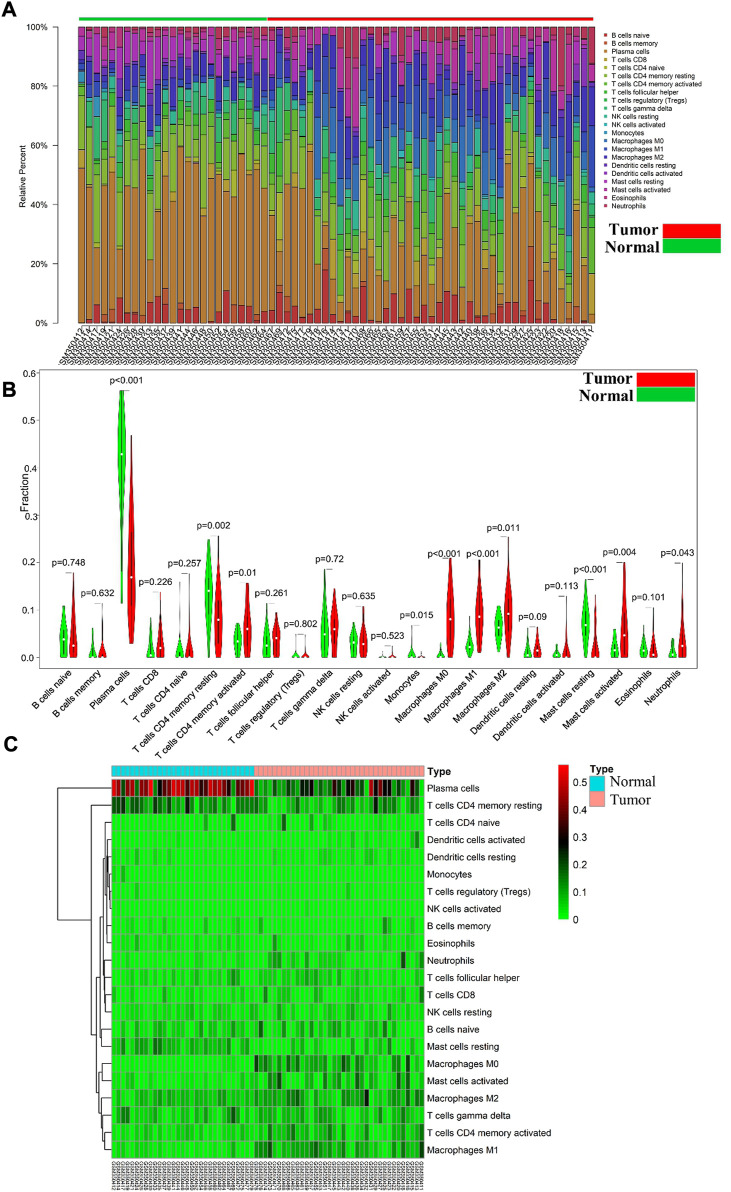
**(A)** Each bar represents a sample, and each color represents a type of immune cell. Area of the color represents the percentage of immune cell infiltration responsible for total immune cell infiltration; **(B)**. Red and green violin columns represent patients with tumor and normal tissues, respectively. The vertical axis represents the ratio of immune cell infiltration responsible for total immune cell infiltration. *p*-value, obtained using the Wilcoxon test, represents the difference between the immune cell infiltration level in patients with VSR and VAF; **(C)**. Each column represents a sample, and each row represents a type of immune cell. Color transition from green to red represents an increase in immune cell infiltration level. Contrast group = normal tissue; trial group = tumor tissue.

**FIGURE 2:**
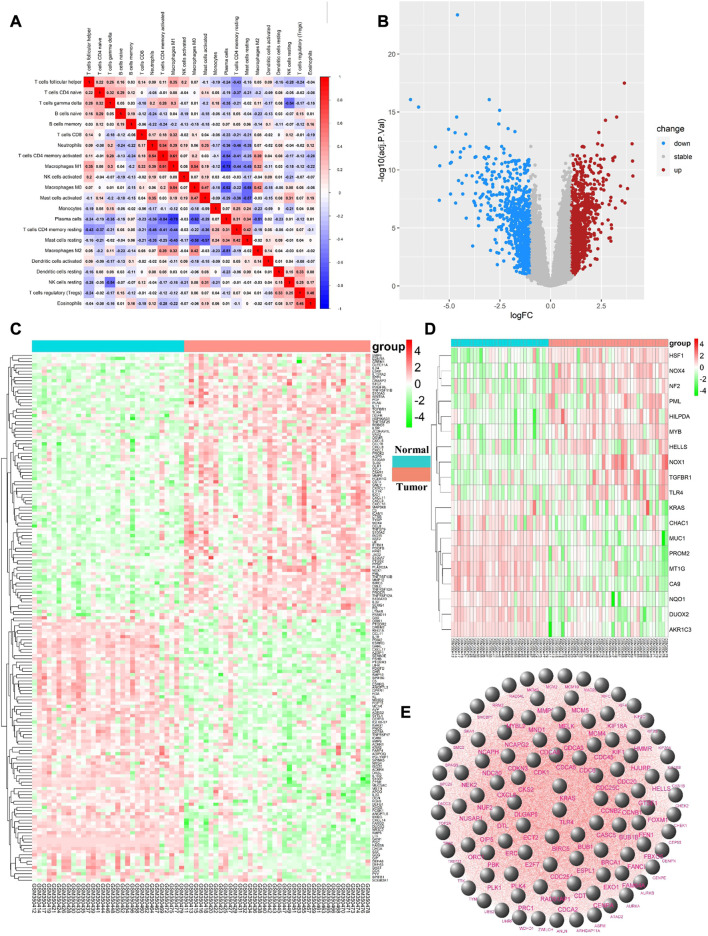
**(A)** the vertical axis and horizontal axis both represent the type of immune cells, and the value in each cell represents the correlation degree of the two immune cells. The higher the correlation, the redder the color; **(B)**. Upregulated DEGs are highlighted in red and downregulated DEGs in blue. Criteria: |logFC| ≥ 1 and adjP value ≤ 0.05; **(C)**. Expression levels of 167 ImmDEGs are shown, the darker the red color, the higher the expression level, and the darker the green color, the lower the expression level. Con group = normal tissue; trial group = tumor tissue; **(D)**. Same as **(C)**, expression levels of 19 FerDEGs are shown; **(E)**. The PPI network was shown.

Differential immune cell infiltrations between normal tissue and gastric cancer tissues was shown in Figure 1A. We then used Wilcoxon test to find that resting CD4 memory T cells (*p* = 0.002), activated CD4 memory T cells (*p* = 0.01), M0 macrophages (*p* < 0.001), M1 macrophages (*p* < 0.001), M2 macrophages (*p* = 0.011), activated mast cells (*p* = 0.004) and neutrophils (*p* = 0.043) showed high levels of infiltration in gastric cancer tissues, while plasma cells (*p* < 0.001), monocytes (*p* = 0.015) and rested mast cells (*p* = 0.001) showed relatively higher levels of infiltration in normal tissues (see Figures 1B, C). Immunocell correlation analysis showed a high correlation between activation phase CD4 memory T cells and M1 macrophages (see Figure 2A). Differential gene expression analysis showed that 1,892 DEGs were obtained, including 1,012 upregulated genes and 880 downregulated genes (see Figure 2B).

### 3.3 Differential expression of ImmDEGs and FerDEGs

167 ImmDEGs was identified by the analysis of immune related gene expression profiles. 84 of these ImmDEGs (e.g., TLR4, CTSB, PGF) were highly expressed and 83 ImmDEGs (e.g., ESRRG, GHRL, CHGA) were lowly expressed in tumor tissues (see Figure 2C). At the same time, analysis of ferroptosis-related genes associated normal tissue and gastric cancer tissues screened out 19 FerDEGs: ten of them, i.e., TLR4, KRAS, HSF1, was highly expressed and nine of them, i.e., MUC1, PROM2, MT1G, were lowly expressed in tumor tissues (see Figure 2D).

### 3.4 PPI network construction, hub gene selection, and enrichment analysis

**FIGURE 3:**
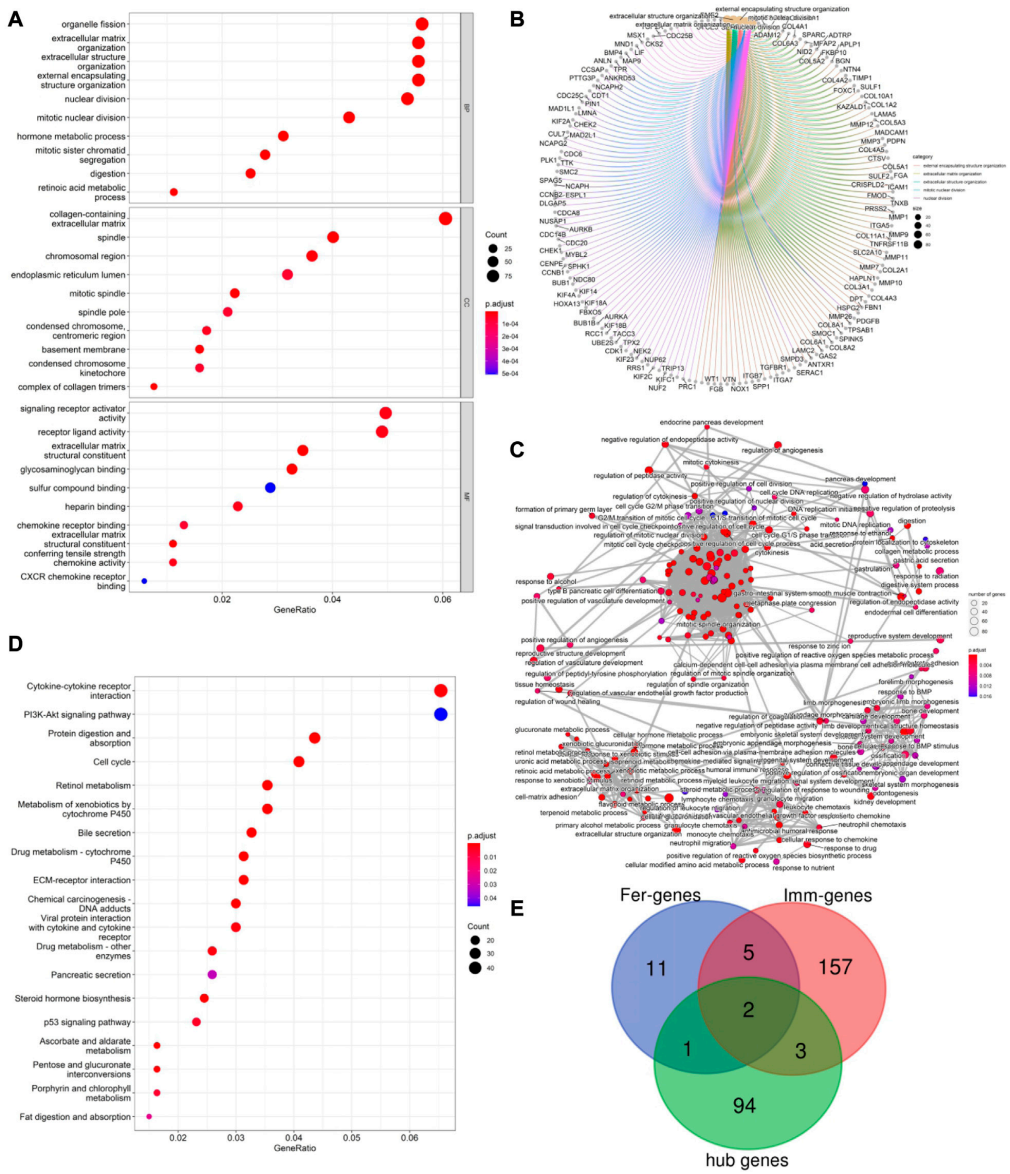
**(A)** Top 10 GO enrichment results were displayed, the horizontal axis represents the gene ratio, i.e., the ratio of the number of DEGs to number of total genes. Dot size is proportional to the gene ratio, and dot color from blue to red that the adjusted *p*-value is smaller; **(B)**. Association among the top 5 immune-related biological processes was identified by analyzing co-enriched DEGs; **(C)**. The relation of interaction between enriched biological process was shown, the circular node represents the biological process, the larger the node size and color are consistent with the enrichment results, the higher the gene ratio is, the larger the node is, and the redder the node color is, the higher the significance is; **(D)**. Same as Figure 2A, KEGG enrichment results were displayed; **(E)**. TLR4 and KRAS at the intersection of 167 ImmDEGs, 19 FerDEGs, and 100 hub genes.

PPI network analysis was conducted on DEGs using string database, with medium confidence ≥ 0.4, and then the top 100 genes were identified as hub genes (see Figure 2E). GO enrichment analysis indicated that DEGs were mainly enriched in 343 biological processes (GO-BP) such as extracellular matrix organization, extracellular structure organization, digestion, retinoic acid metabolic process, hormone metabolic process. The DEGs were enriched in 37 cellular components (GO-CC) including complex of collagen trimer, basement membrane, endoplasmic reticulum lume, and were enriched in 35 molecular functions (GO-MF) such as signal receptor activator activity, receptor ligand activity, chemokine activity, chemokine receptor binding (see Figure 3A). Furthermore, by analyzing co-enriched DEGs, some relationships between immune-related BPs, and some common DEGs between immune-related BPs were screened out by us such as BGN and MFAP2. These DEGs were co-enriched in several different BPs and get involved in external encapsulating structure organization, extracellular matrix organization as well as extracellular structure organization (Figure 3B). In fact, many biological processes are mutually interacting such as cellular response to chemokine, neutrophil chemotaxis and neutrophil migration (see Figure 3C). In terms of KEGG pathway enrichment analysis, 19 pathways such as cytokine-cytokine receptor interactions, retinol metabolism, and protein digestion and absorption were enriched (Figure 3D).

### 3.5 Identification of key genes and prediction of herbal medicine

By taking the intersection of ImmDEGs, FerDEGs and hub genes, two common key genes, TLR4 and KRAS were identified (see Figure 3E). 12 herbs (e.g., *Rhizoma Coptidis, Folium Isatidis, Acidum Citricum, Radix Stellariae, Trifolium fragiferum*) and 27 ingredients (e.g., resveratrol, cinnamaldehyde, oleuropein, shikonin, magnolol) were predicted the HERB database base on this two key genes (Tables 2, 3).

**TABLE 2:** Herbal medicines predicted on the basis of key genes.

Key genes	KRAS		TLR4
Herbal medicines	Rhizoma Coptidis	Diospyros kaki	Radix Salviae liguliobae
	Folium Isatidis	Rhizoma Polygoni cuspidati	Rhizoma Coptidis
	Acidum Citricum	Scutellaria epilobifolia	
	Radix Stellariae	Sinocalamus oldhami	
	Trifolium fragiferum	Anodendron affine	

**TABLE 3:** Herbal ingredients predicted on the basis of key genes.

Key genes	TLR4		KRAS	
Herbal ingredients	Resveratrol	Gedunin	Carnosine	Magnolol
	Cinnamaldehyde	Curcumin	Vitisin b	Rasfonin
	Oleuropein	Dioscin	Coumarin	Rocaglamide
	Shikonin	Taxol	Flavonol	
	Stigmasterol	Puerarin	Ursolic acid	
	Pinocembrin	Punicalagin	Prostratin	
	Nordihydroguaiareticacid	Saikosaponin a	n-acetyl-d-glucosamine	
	Tauroursodeoxycholic acid	Salvianolic acid b	Glycyrrhetinic acid	

### 3.6 Molecular docking

**FIGURE 4:**
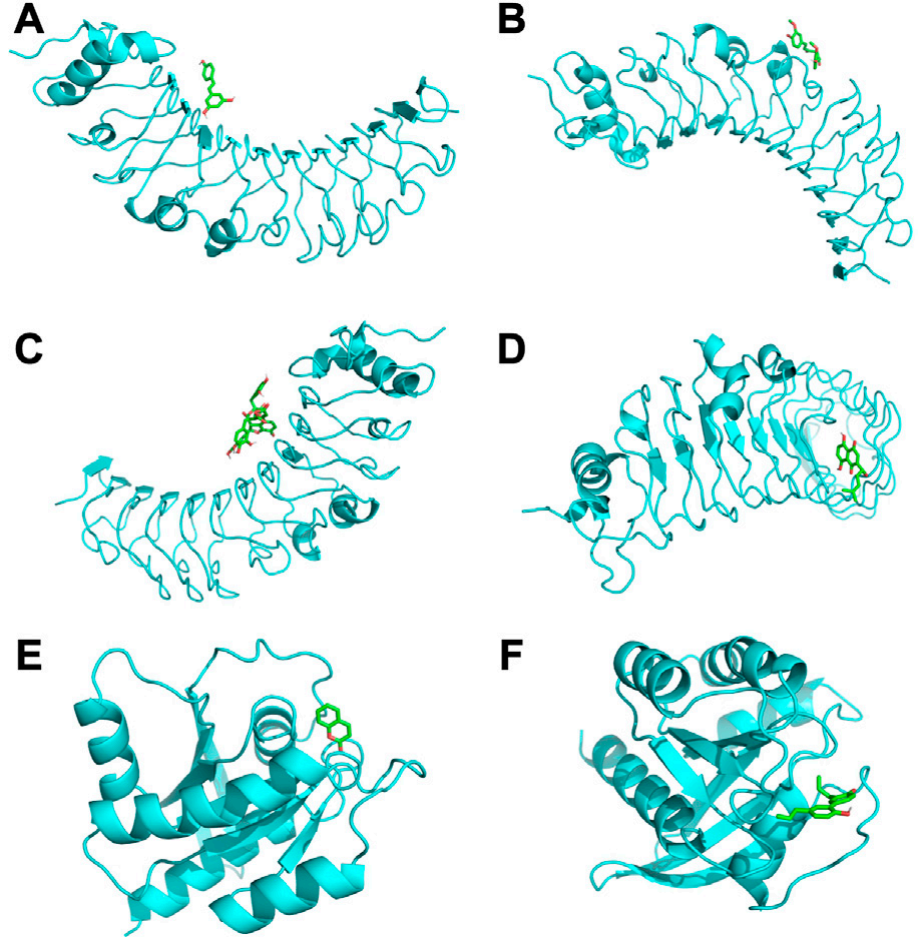
**(A)** shows TLR4 docked with resveratrol (−6.2 kcal/mol); **(B)** shows TLR4 docked with curcumin (−5.9 kcal/mol); **(C)** shows TLR4 docked with salvianolic acid b (−7.6 kcal/mol); **(D)** shows TLR4 docked with shikonin (−6.9 kcal/mol); **(E)** shows KRAS docked with coumarin (−6.7 kcal/mol); **(F)** shows KRAS docked with magnolol (−6.9 kcal/mol).

Among the molecular docking results of these herbal ingredients with key genes (Figures 4A–F), we found that TLR4 could bind closely to resveratrol (−6.2 kcal/mol), curcumin (−5.9 kcal/mol), salvianolic acid b (−7.6 kcal/mol), and shikonin (−6.9 kcal/mol), while KRAS bound closely to coumarin (−6.7 kcal/mol) and magnolol (−6.9 kcal/mol).

### 3.7 Expression of key genes after resveratrol and magnolol intervention detected by quantitative reverse transcription PCR

**FIGURE 5:**
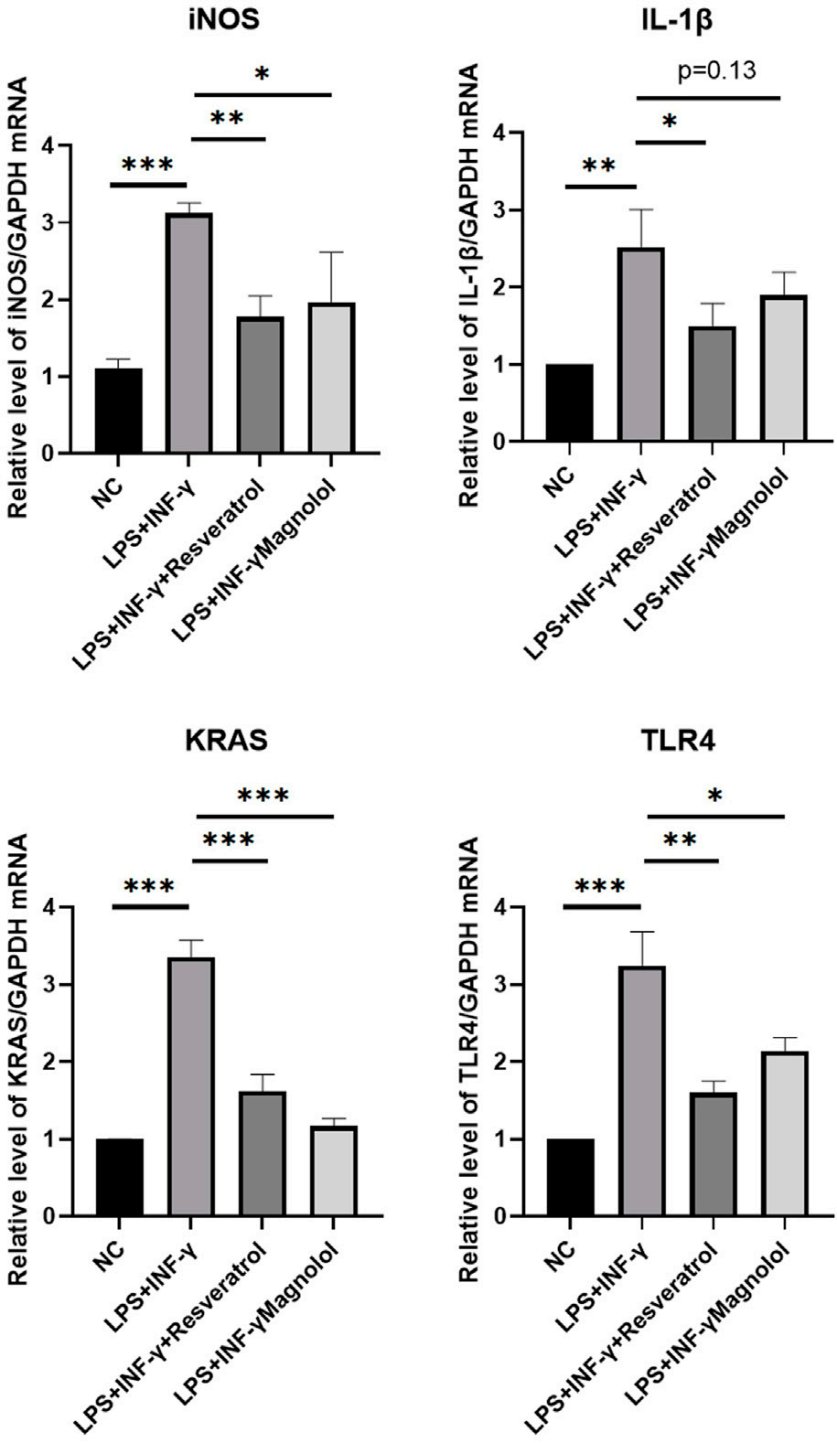
RT-QPCR results for validation over three independent experiments. Data are shown as mean ± standard error of the mean. *p < 0.05, **p < 0.01, ***p < 0.001.

The results revealed that stimulation with LPS + IFN-γ substantially elevated the mRNA levels of iNOS, IL-1, KRAS, and TLR4 compared to NC group (Figure 5). Both resveratrol and magnolol administration markedly decreased the amount of key gene expression. Resveratrol and magnolol significantly reduced the expression of IL-1, iNOS, KRAS, and TLR4 as compared to the LPS + IFN-γ group.

## 4 Discussion

Gastric cancer is one of the leading causes of cancer deaths worldwide, with over one million new cases diagnosed each year (Smyth et al., 2020). The rate of early diagnosis of gastric cancer is low and survival time for patients with advanced metastases is usually less than 1 year (Patel and Cecchini, 2020). Surgical resection of the cancer is the accepted first line of treatment. However, although the 5-year survival rate for patients who undergo surgery at an early stage can be as high as 95% (Song et al., 2017), the recurrence rate after surgery is high, even if the cancer site is completely removed (Johnston and Beckman, 2019). Hence, there is an urgent demand to find novel mechanisms involved in gastric cancer, which may provide promising perspectives on treatment of gastric cancer patients. Ferroptosis and tumor immune micro-environment are one of the key mechanisms of gastric cancer.

Ferroptosis is a regulated form of cell death characterized by the iron-dependent accumulation of lipid hydroperoxides to lethal levels. Ferroptosis is associated with cellular vital metabolism in many diseases, including neoplastic diseases such as gastric cancer and non-neoplastic degenerative diseases such as Alzheimer’s disease, Parkinson’s disease, and ischemia-reperfusion injury (Stockwell et al., 2017; Chen et al., 2021b). Because of the specificity and breadth of this regulated cell death, interventions employing ferroptosis gene target agonists to induce ferroptosis in cancer cells are thought to have great anti-cancer potential (Lee et al., 2020). In this study, we screened 19 genes associated with ferroptosis, of which 10 FerDEGs such as TLR4, KRAS and HSF1 were highly expressed and 9 FerDEGs such as MUC1, PROM2 and MT1G were lowly expressed in tumour tissue. All of these targets have great potential for further study. However, among these genes, TLR4, a member of the TOLL-like receptor family, is the most interesting target among these FerDEGs. TLR4 is the target of intervention by multiple mechanisms, including ferritinases, inflammation and autophagic pathways. Thus, intervention on TLR4 may simultaneously produce therapeutic effects from multiple parallel pathways, which has more potential than other single pathway targets of intervention.

Disturbances in the immune system are one of the key causes of tumour development. When tumour cells develop, immune evasion due to immune imbalance causes tumour cells to become unrestrained and uncontrolled. In this study, immune infiltration analysis showed that resting CD4 memory T cells, activated CD4 memory T cells, monocytes, M0 macrophages, M1 macrophages, M2 macrophages, activated mast cells and neutrophils exhibited high levels of infiltration in gastric cancer tissues. There is growing evidence that aberrant behaviour of innate and adaptive immune cells in the tumour microenvironment (TME) contributes to tumour progression (Gajewski et al., 2013). Thus, as shown by the results of immune cell correlation analysis, the significant correlation between activated CD4 memory T cells (adaptive immune cells) and M1 macrophages (innate immune cells) is a manifestation of innate and adaptive immune interactions. Indeed, immune cells themselves are an important component of the tumour stroma and cancer cells themselves can interact with proximal immune cells by secreting various cytokines and chemokines, resulting in TME that is more conducive to tumour growth and metastasis (Li et al., 2020).

Interestingly, there is crosstalk between the immune response and ferritin formation, a property that has been applied in tumour therapy, for example, using CD8^+^ activation to induce ferritin formation in tumour cells (Tang et al., 2020). This crosstalk can occur in 2 ways: the immune cells themselves undergo ferroptosis in immune-disordered TME; immune cells recognise the tumour cells undergoing ferroptosis and generate an inflammatory clearance response (Hsu et al., 2021). It is clear that we expect a second metabolic modality to occur in tumour cells in TME, and although the exact details of the crosstalk between ferroptosis and immune disorders are currently poorly understood in humans, targeting and modulating this metabolic modality in tumour cells in TME is a very promising therapeutic approach. We therefore hope to predict some of the drugs that regulate this complex metabolic pathway by searching for genes common to both immunity and ferroptosis.

This study identified TLR4 and KRAS as the key genes through a comprehensive analysis of immune infiltration-related and ferroptosis-related genes. TLR4 has been associated with cancer in multiple ways. Various cell lines and tissue samples from patients with head and neck, esophageal, gastric, colorectal, liver, pancreatic, skin, breast, ovarian, cervical, and breast cancer were found to express high levels of TLR4 (Awasthi, 2014). TLR4 has been shown to be involved in immune responses to inflammatory signaling pathways that promote gastric carcinogenesis and to inhibit the immune response by mediating macrophage M2-type polarization, promoting tumor cell metastasis and being strongly associated with the outlook for those diagnosed with gastric cancer (He et al., 2018; Li et al., 2021). Activation of TLR4-NF-B under inflammatory conditions induces cancer cell motility and invasion (Liao et al., 2012; Huang et al., 2020a). Inhibition of TLR4 by siRNA and NF-B inhibitors can reduce cancer cell invasiveness. TLR4 induces an effective cytotoxic T cell immune response to tumour antigens (Fang et al., 2014). Ultimately, cytotoxic T cells will eliminate cancer cells. Numerous studies have demonstrated that TLR4 agonists do not significantly induce ferroptosis in cancer cells, but rather cause a negative immune response in in vivo against tumour population cells due to increased ferroptosis, indirectly creating a TME that is more favourable to cancer cell proliferation (Shetab Boushehri and Lamprecht, 2018; Kashani et al., 2021). Inhibition of TLR4 significantly attenuates the proliferation of inflammatory tumour cells, a phenomenon that has been demonstrated in several studies, such as the inhibition of the expression of this gene impedes the growth of colitis-associated colon and ovarian cancer cells (Earl et al., 2009; Kashani et al., 2020). Inhibition of TLR4 has been shown to reduce tumour burden in mouse models of hepatic steatosis and colorectal metastasis (Timar and Kashofer, 2020). Therefore, we suggest that TLR4 may be an important target in gastric cancer TME, where immune responses and ferritinase interactions are probably based on this key genes.

RAS mutation represents the most common oncogenic changes in human tumours. KRAS is the subtype of RAS that mutates at the highest rate (Quinlan et al., 2008; Fan et al., 2021). The proliferation of endothelium stem and progenitor cells is controlled by activated KRAS, which also plays a role in promoting the development of endodermally derived cancers. Oncogenic KRAS is responsible for cancer cell proliferation, energy metabolism, chemoresistance, invasion and metastasis (Fisher et al., 2001). KRAS extinction has been shown to cause tumour regression in genetically produced KRAS-mutant cancer (Boutin et al., 2017; Ayatollahi et al., 2018). Approximately to 11% of gastric cancer (GC) patients have KRAS mutations, with the most prevalent mutations occurring at codons 12 and 13 (Hewitt et al., 2019). Furthermore, according to a large multicenter investigation, KRAS activation occurs in the early phases of GC before developing a phenotype (Qian et al., 2014). KRAS activation is associated with EMT, MSI status and poor patient outcomes (Fu et al., 2019; Polom et al., 2019). Within malignancies, KRAS may be responsible for generating an inflammatory environment. Cancer cells driven by KRAS release cytokines and other substances to regulate stromal cells, such as fibroblasts and innate and adaptive immune cells, in their vicinity. Activation of the KRAS signaling pathway may stimulate the synthesis of chemokines, such as interleukin-8 (CXCL-8/IL-8), LIX, MIP2, KC and MCP-1 (Sparmann and Bar-Sagi, 2004; Ji et al., 2006). Positive feedback loops stimulate inflammatory mediators Src, EGR1, STAT3, NF-kB, and Cox-2 to maintain the oncogenic activity of KRAS (di Magliano and Logsdon, 2013). The mutant KRAS secretes IL-10 and TGF-b1 *via* the MEK-ERK-AP1 pathway, therefore inhibiting the activation of T cells (Zdanov et al., 2016). Immune evasion is a defining feature of KRAS-driven cancer, whereas KRAS produces immunosuppressive effects in tumour tissue by promoting macrophage M2 polarisation (suppressing-inflammatory phenotype), thereby creating a more hospitable TME ecological niche for cell proliferation and metastasis (Ischenko et al., 2021). It has been shown that KRAS-G12D protein released from tumour cells undergoes ferrogenesis and is taken up by macrophages, leading to the conversion of macrophages to an M2-like suppressing-inflammatory pro-tumour phenotype (Janes et al., 2018). Not surprisingly, KRAS acts as a dual regulator of the immune response and ferroptosis, similar to the role of TLR4 described above. Although KRAS was previously thought to be untargetable, studies coming in have found that targeted inhibitors of KRAS work satisfactorily (Fang et al., 2021). Therefore, herbal medicines and herbal ingredients predicted on the basis of KRAS also have great potential.

The HERB database is the world’s largest and most advanced database for the modernisation of Chinese medicine. The data in the database are derived from high-throughput experiments, so the database predicts results with high reliability (Fu et al., 2022). In this study, a total of 12 herbal medicines such as *Rhizoma Coptidis, Folium Isatidis, Acidum Citricum, Radix Stellariae, T. fragiferum* and 27 herbal ingredients such as resveratrol, cinnamaldehyde, oleuropein, shikonin and magnolol, were directly predicted based on the key genes TLR4 and KRAS. In fact, some of the predicted herbs such as *Rhizoma Coptidis* (Chai et al., 2018; Kim et al., 2019), *Rhizoma Polygoni cuspidati* (Jiang et al., 2017) and *Radix Salviae liguliobae* (Wilke et al., 2014) have been shown to have significant anti-gastric cancer effects. Predicted anti-gastric cancer effects of herbal ingredients such as *taxol* (Huang et al., 2020b; Zhang et al., 2021), *coumarin* (Kaur et al., 2015; Xu et al., 2017), *resveratrol* (Yan et al., 2020; Ren et al., 2021) and *magnolol* (Zhao et al., 2022) have also been demonstrated. Among the molecular docking results of these herbal ingredients with key genes, we found that TLR4 could bind closely to salvianolic acid b (−7.6 kcal/mol), and shikonin (−6.9 kcal/mol), while KRAS bound closely to coumarin (−6.7 kcal/mol) and magnolol (−6.9 kcal/mol). Salvianolic acid B have potent efficacy on cancer *in vitro* and *in vivo*. The mechanisms mainly include induction of apoptosis, autophagy and cell cycle arrest, anti-metastasis, formation of cancer stem cells, and potentiation of antitumor immunity (Boulos et al., 2019). At low doses, Shikonin produced apoptotic cell death and at high amounts, necroptosis (Xuan and Hu, 2009; Yang et al., 2014). Shikonin triggered dose-dependent apoptosis in cancer cells by raising intracellular levels of reactive oxygen species (ROS), which are primarily formed by lipoxygenase, NADPH oxidase, and mitochondrial complex II (Zhao et al., 2022).

Finally, Experiments *in vitro* were used to verify the modulatory effect of resveratrol and magnolol on immune imbalance after THP-1 cell stimulating with LPS + IFN-γ. We examined the expression levels of key genes (iNOS, IL-1, KRAS and TLR4) in each macrophage group by quantitative reverse transcription PCR and found that the expression levels of these genes in the resveratrol and magnolol groups were significantly lower than those in the LPS + IFN-γ group (see Figure 5).

Our study’s findings indicated that resveratrol and magnolol, which have both therapeutic and preventative benefits for patients with gastric cancer, have significant clinical implications. Resveratrol, magnolol, and our study model offer considerable potential in this field, especially in light of the apparent underdevelopment of treatments for gastric cancer. With the use of immune infiltration and ferroptosis, this study introduced a new research approach for predicting potential therapy options for gastric cancer, paving the way for the development of novel therapeutic approaches.

Therefore, these predicted herbs and herbal ingredients have great potential to improve the prognosis of gastric cancer patients by acting on the targets TLR4 and KRAS, and provide alternative options and direction for anti-gastric cancer herbal research.

## Conclusion

Immune evasion is one of the major causes of loss of restraint in tumour cells, and iron death is an important modality regulating tumour cell death. TLR4 and KRAS, as common genes for immune infiltration and ferroptosis, are involved in gastric carcinogenesis and play an important role in gastric cancer progression. Based on the prediction of these two key genes, herbal ingredients such as resveratrol, magnolol, curcumin, salvianolic acid b, as well as herbal medicines such as *S. liguliobae, Rhizoma Coptidis* and *Rhizoma Polygoni cuspidate* provide research directions and alternative therapies for immunomodulation of TME and ferroptosis of tumour cells in gastric cancer.

## Data Availability

The datasets presented in this study can be found in online repositories. The names of the repository/repositories and accession number(s) can be found in the article/Supplementary Material.
